# LINC00665 promotes the progression of acute myeloid leukemia by regulating the miR-4458/DOCK1 pathway

**DOI:** 10.1038/s41598-021-82834-9

**Published:** 2021-03-03

**Authors:** Xiaoyu Yang, Yan Wang, Sulei Pang, Xiaojie Li, Panpan Wang, Ruojin Ma, Yunyun Ma, Chunge Song

**Affiliations:** Department of Hematology, The Fifth Affiliated Hospital of Zhengzhou University, No.3 Kangfu Front Road, ZhengzhouHenan, 450052 China

**Keywords:** Cancer genetics, Cancer, Cell biology, Genetics, Molecular biology

## Abstract

This study aimed to explore the role of LINC00665, miR-4458 and DOCK1 and their interactions in the development of acute myeloid leukemia (AML). The relative expression of LINC00665, miR-4458 and DOCK1 in AML samples was measured using qRT-PCR, and the protein level of DOCK1 in AML cell lines was examined using western blot. CCK8, BrdU, transwell, cell adhesion, and caspase-3 activity assays were carried out to evaluate the viability, proliferation, migration, adhesion, and apoptosis of AML cells, respectively. Luciferase reporter, RIP, and RNA pull-down assays were also performed to confirm the target relationship among LINC00665, miR-4458 and DOCK1. Findings revealed that LINC00665 and DOCK1 were aberrantly overexpressed in AML tissues and that the expression of miR-4458 was low in AML tissues. Silencing LINC00665 or DOCK1 presented significant restriction to the proliferation, migration and adhesion of AML cells. Apart from that, it was found that inhibiting miR-4458 could enhance the proliferation, migration and adhesion of AML cells but suppress the apoptosis of AML cells. Experimental results also indicated that LINC00665 exerted its positive function on AML cells by sponging miR-4458 and that miR-4458 influenced the progression of AML cells by targeting DOCK1 directly. Overall, this finding not only provided a novel molecular pathway for the diagnosis and treatment of AML but also showed that LINC00665 could enhance the progression of AML by regulating the miR-4458/DOCK1 pathway.

## Introduction

Acute myeloid leukemia (AML) is a kind of hematological malignancy caused by abnormal proliferation and differentiation of myeloid hematopoietic progenitor cells^1^. AML progresses rapidly in the body. Simply put, the aberrantly proliferative and differentiated myeloid cells infiltrate into blood, marrow blood and other tissues, leading to impaired hematopoiesis and a variety of complications (i.e., systemic infection) that eventually threaten patients’ life^2, 3^. AML is the most prevalent type of leukemia among adults, with nearly 20,000 new adult cases annually across the globe^4^. Although the continued progress of chemotherapy and stem cell transplantation has significantly improved AML prognosis, the survival rate of AML patients, especially those over 60 years old, is still very low due to the chemotherapy resistance and high recurrence rate of this malignant growth^5–7^. Therefore, it is still crucial to understand the pathogenesis of AML and explore new therapeutic targets for AML.

Long non-coding RNAs (lncRNAs) refer to a class of non-coding transcripts with a length of over 200 nucleotides^8, 9^. Even though lncRNAs have no protein-coding capacity, they participate actively in the progression of various malignancies, including AML, by modulating the expression of microRNAs^10–12^. For instance, lncRNA C5orf66-AS1 was reported to promote cell proliferation in cervical cancer by targeting the miR-637/RING1 axis^13^. In one research, lncRNA HOXA-AS2 was discovered to reduce the chemoresistance of AML cells by sponging miR-520c-3p to regulate S100A4 expression^14^. Overexpressed lncRNA linc-223 in AML cells was also demonstrated to induce cell cycle arrest and differentiation of monocytes by downregulating miR-125-5p, which further targeted the interferon regulatory factor (IRF4)^15^. LINC00665, a lncRNA, was recently identified and suggested to play a regulatory role in the tumorigenesis of breast cancer^16^, gastric cancer^17^ and prostate cancer^18^ by sponging microRNAs. Even though these findings highlighted the potential value of LINC00665 in cancer therapy, it remains unknown whether and how LINC00665 could regulate the progression of AML.

Regarded as small non-coding RNAs, microRNAs (miRNAs) have drawn research interests in the modulation of multiple carcinogenesis^19, 20^. Several studies reported that miR-4458 served as a tumor suppressor in the progression of many types of cancers. For example, miR-4458 was found to inhibit cell proliferation and promote cell apoptosis by targeting SOCS1 in triple-negative breast cancer^21^. One study on breast cancer documented the suppressive effect of miR-4458 on the growth and migration of tumors via the mediation of CPSF4^22^. Moreover, miR-4458 was reported to contribute to the aberrant proliferation and migration of cancer cells by sponging several lncRNAs such as CDKN2B-AS1 and KCNQ1OT1^23, 24^. Nonetheless, the function of miR-4458 and its potential sponger in AML are yet to be well understood.

The dedicator of cytokinesis 1 (DOCK1), also known as DOCK180, can be described as a member of atypical Rho guanine nucleotide exchange factors (GEF) family, and it has been reported to participate in biological and pathological processes such as cell cycle, cell migration, phagocytosis and tumorigenesis^25–28^. DOCK1 was also reported to be highly expressed in AML patients with poor prognosis, meaning it could act as an oncogene or aggravate the progression of AML^29^. Nevertheless, the exact function of DOCK1 in AML pathogenesis is still unknown. Our objective in this research was to explore the role of LINC00665, miR-4458 and DOCK1 and their interactions in the tumorigenesis of AML. We believed that this research could be relevant in that it might provide new clues for developing novel strategies for AML diagnosis and therapy.

## Materials and methods

### Bioinformatics analysis

GEPIA, an online tool, was utilized to identify differentially expressed genes (DEGs) and AML survival-associated genes. Apart from that, STRING was used to identify the key gene by constructing the protein–protein interactions. The ENCORI starBase and TargetScan algorithms were employed to predict the miRNAs that could bind to LINC00665 and DOCK1 mRNA, respectively. Finally, Venny 2.1.0 was applied to overlap the genes and miRNAs.

### Clinical samples collection

Bone marrow specimens were collected from 36 AML patients and 36 healthy donors (patients without AML). The participants underwent bone marrow aspiration and biopsy examination at The Fifth Affiliated Hospital of Zhengzhou University. All the recruited AML patients were diagnosed with the French-American-Britain (FAB) criteria. The clinical characteristics of 36 cases of AML are shown in Table 1. All the donors were mandated to sign the informed consent form before sample collection. The study was performed with the approval of the Ethical Committee of The Fifth Affiliated Hospital of Zhengzhou University. The processing of clinical tissue samples is in strict compliance with the ethical standards of the Declaration of Helsinki.

**Table 1:** Clinical characteristics of 36 cases of acute myeloid leukemia patients.

Characteristics	Total = 36	Percentage (%)	
**Age (years)**			
> 45	17	47.2	
≤ 45	19	52.8	
**Gender**			
Male	20	55.6	
Female	16	44.4	
**FAB subtypes**			
M0	2	5.6	
M1	7	19.4	
M2	9	25.0	
M3	3	8.3	
M4	5	13.9	
M5	7	19.4	
M6	1	2.8	
M7	0	0.0	
Unclassified	2	5.6	
**Cytogenetic risk group**			
Favourable	9	25.0	
Intermediate	17	47.2	
Unfavourable	10	27.8	
**White blood cell (× 10**^**9**^**/L)**			
Median (range)	24.5 (1.2–309.8)		
**Hemoglobin (g/dL)**			
Median (range)	8.78 (6.8–12.2)		
**Platelet (× 10**^**9**^**/L)**			
Median (range)	38.5 (2.2–718.7)		

*FAB* French–American–Britain.

### Cell lines and culture

Human AML cell lines (KG1, U937, NB4 and HL60) and normal bone marrow cell line (HS-5) were bought from the American Type Culture Collection (ATCC, USA). The Roswell Park Memorial Institute (RPMI)-1640 medium provided by Gibco (USA) was supplemented with 10% fetal bovine serum, 100 µg/ml streptomycin and 100 U/ml penicillin to culture all the cell lines with 5% CO_2_ at 37 °C.

### RNA extraction and quantitative real-time PCR (qRT-PCR)

All the RNAs were extracted from AML or normal bone marrow tissues and cells with the TRIzol reagent (Invitrogen, USA). After quantifying and assessing the RNAs with NanoDrop 2000 (Thermo Fisher Scientific, USA), the All-in-One miRNA qRT-PCR Detection Kit (GeneCopoeia, China) was utilized to examine miR-4458 expression. As for the measurement of LINC00665 and DOCK1 mRNA expression, the PrimeScriptVR RT reagent Kit (Takara, Japan) was applied to reverse-transcribe PCR and generate cDNA. Then, the SYBR Premix Ex Taq (Takara, Japan) was used qRT-and subjected to qRT-PCR was conducted through ABI 7900 system (Thermo Fisher Scientific, USA). The relative expression of LINC00665 and DOCK1 were subsequently normalized by GAPDH, while that of miR-4458 was normalized by U6. The 2^−ΔΔCT^ method was used to calculate their expression levels. The primer sequences are illustrated in Table 2.

**Table 2:** The primer sequences for RT-qPCR.

GENE	Primer sequences (5′–3′)
miR-4458	Forward: AGAGGTAGGTGTGGAAGAA
	Reverse: GCGAGCACAGAATTAATACGAC
U6	Forward: CTCGCTTCGGCAGCACA
	Reverse: AACGCTTCACGAATTTGCGT
LINC00665	Forward: GGTGCAAAGTGGGAAGTGTG
	Reverse: CGGTGGACGGATGAGAAACG
DOCK1	Forward: CCGCCGCAAACTTTTTCCTC
	Reverse: AGATGTGCACAGTGTCTCCG
GAPDH	Forward: AGCCACATCGCTCAGACAC
	Reverse: GCCCAATACGACCAAATCC

### Cell transfection and treatment

The LINC00665 siRNA, miR-4458 mimics, miR-4458 inhibitor, DOCK1 siRNA, pcDNA3.1-DOCK1 overexpression vector and negative control were purchased from RiboBio (China) for cell transfection. 1 × 10^6^ or 5 × 10^3^ U937 and HL60 cells were seeded into each well of the 6-well plates or 96-well plates. After incubating overnight with 5% CO_2_ at 37 °C, LINC00665 siRNA (si-LINC), miR-4458 mimics, miR-4458 inhibitor, DOCK1 siRNA (si-DOCK1) and negative control, pcDNA3.1-DOCK1 overexpression vector or pcDNA3.1 empty vector were transfected into U937 and HL60 cells with Lipofectamine 2000 Transfection Reagent (Invitrogen, USA). The sequences of these vectors are listed in Supplementary Table 1. Six hours later, the culture medium was pipetted out and replaced with a new medium. After 48 h of transfection, the transfected cells were harvested for further assay. The Rac1 inhibitor was purchased from Sigma-Aldrich (USA), and the Rac1 inhibitor treatment was performed at the transfection meantime with a concentration of 50 μM.

### Luciferase reporter assay

Human LINC00665 segments and DOCK1 mRNA 3′ UTR containing predicted sequences interacting with miR-4458 were amplified and cloned into the psiCHECK-2 luciferase vector (Promega, USA), and the incorporated constructs were named LINC00665-wt and DOCK1-wt, respectively. With reference to the construction of mutant reporter plasmids, LINC00665 segments and DOCK1 mRNA 3′ UTR with a site mutation were synthesized and cloned into the psiCHECK-2 luciferase vector and then named LINC00665-mut and DOCK1-mut, respectively. Next, these luciferase reporter plasmids were transiently transfected into U937 and HL60 cells along with the miR-4458 mimic, miR-44582 inhibitor or negative control. After culturing for 48 h, the culture medium was collected and incorporated into the dual-luciferase reporter assay system (Promega, USA) to detect the luciferase activity.

### RIP assay

The EZ-Magna RIP RNA-binding protein immunoprecipitation kit (Millipore, USA) was used in the RIP assay to determine the interaction between LINC00665 and miR-4458 in U937 and HL60 cells. Briefly, 1 × 10^7^ U937 and HL60 cells were harvested and lysed with the lysis buffer. After that, the cell lysates were incubated at 4 °C for 2 h with the anti-human Ago2 antibody (Millipore, USA) coated with magnetic beads. The anti-human IgG was used as the control. The magnetic beads were washed with the RIP buffer three times and then washed again with PBS. The precipitated RNAs were subsequently isolated from the resuspending beads with the TRIzol reagent. After that, they were subjected to LINC00665 and miR-4458 expression measurement using qRT-PCR analysis.

### Cell viability assay

The cell viability of U937 and HL60 cell lines were evaluated using the Cell Counting Kit-8 (CCK-8) (Biotool, USA) according to the manufacturers’ guidelines. Briefly, the transfected cells were plated into 96-well plates at a density of 3 × 10^3^ cells/well. After 0 h, 24 h, 48 h and 72 h of incubation at 37 °C in an atmosphere containing 5% CO_2_, 10 μL CCK-8 solution was pipetted into the cell wells and cultured for another 2 h. The optical density (OD) at 450 nm was finally measured with a microplate reader (Bio-Rad, USA).

### Cell proliferation assay

This assay was performed with the BrdU Cell Proliferation ELISA Kit (Abcam, USA). According to the manufacturers’ instructions, the transfected cells were harvested and transferred into 96-well plates at a density of 2 × 10^5^ cells/well. 20 μL/well diluted 1X BrdU labeling solution was added to the 96-well plates. After incubating for 2 h at 37 °C, the medium was removed. Next, 200 μL/well Fixing Solution was supplied to the cells, and the mixture was incubated at room temperature for 30 min. Then, the plate was washed three times with 1X Wash Buffer, which was supplemented with 100 μL Anti-BrdU Antibody solution, and incubated for 1 h at room temperature. After washing the cells three times, the cells were incubated with 100 μL Peroxidase Goat Anti-Mouse IgG Conjugate at room temperature for 30 min. Subsequently, 100 μL TMB substrate solution was added to each well and incubated for another 30 min at room temperature. Finally, the OD at 450 nm was tested and measured with a microplate reader (BioRad, USA).

### Caspase‑3 activity assay

The caspase-3 activity of U937 and HL60 cells was evaluated with the Caspase-3 Colorimetric Assay Kit (Medical and Biological Laboratories, Japan). This evaluation was done according to the manufacturer’s instructions. Next, 2 × 10^5^ transfected cells were harvested and lysed. After that, 50 μL cell lysates were removed and added to the 96-well plates. Subsequently, 50 μL reaction buffer and 5 μL caspase-3 substrate were added to each well. After incubating for 1 h at 37 °C, the OD at 405 nm was measured with a microplate reader (Bio-Rad, USA). The relative enzymatic activity of the caspase-3 in relation to the blank group was calculated and used for statistical analysis.

### Transwell migration assay

This assay was used to evaluate the migration ability of U937 and HL60 cells. Briefly, the transfected U937 and HL60 cells were harvested, centrifuged and resuspended with the RPMI-1640 medium supplemented with 1% fetal bovine serum. The cell density was first adjusted to 2 × 10^5^ cells/mL. Next, the top chamber of the 24-well plates was plated with 200 μL cell suspension and 600 μL 10% serum-supplemented medium was added to the bottom of the chamber. After culturing for 48 h, the cells migrating to the bottom medium were collected and counted with the blood cell counting plate. Meanwhile, the cells at the top of the chamber were removed, while the attached cells under the chamber were fixed with 4% paraformaldehyde and stained with 0.1% crystal violet solution. Eventually, the images of the attached cells were captured with an inverted microscope camera, and the cell number was counted in each chamber.

### Cell adhesion assay

Before the commencement of this assay, the 96 well plates were first coated with 50 μL of 10 μg/mL type I collagen (BD Bioscience, CA) or 10% bovine serum albumin (BSA, Sigma, USA). After an incubation period of 1 h at 37 °C, the wells were cleaned with PBS and plated with transfected U937 and HL60 cells at a density of 5 × 10^3^ cells/well. Then, the cells were cultured for 1 h at 37 °C. Following that, the culture medium was removed, and the cell wells were carefully rinsed with PBS to remove the non-attached cells. The adherent cells were subsequently fixed with 4% paraformaldehyde, stained with 0.5% crystal violet, and extracted with sodium citrate methanol solution. Finally, the OD at 570 nm was examined with a microplate reader (BioRad, USA), and the ratio of the adhesion ability to the blank group was calculated and utilized for statistical analysis.

### RNA pull-down assay

The RNA pull-down assay was carried out as previously described^30^. Briefly, U937 and HL60 cells at a density of 6 × 10^5^/well were seeded into 6-well plates. After incubating overnight at 37 °C with 5% CO_2_, the cells were transfected with the biotinylated-miR-4458 mimics and biotinylated-negative control designed and synthesized by RiboBio (Guangzhou, China). This transfection was done with Lipofectamine 2000 Transfection Reagent (Invitrogen, USA). After 48-h transfection, the cells lysates were collected, sonicated, and incubated with streptavidin beads (Life Technologies, USA) for 3 h at 4 °C. After washing thrice with PBS, the RNeasy Mini Kit (QIAGEN) was used to elute the bound RNAs. The eluted RNAs were finally used to measure the relative expression of DOCK1.

### Western blot

All the proteins were obtained from AML tissues and cells using the RIPA lysis buffer and were quantified with the Pierce BCA Protein Assay Kit (Thermo Fisher Scientific, USA). SDS-PAGE (8%) was used to separate the protein, and the concentration of loaded protein samples was 40 μg/lane. After performing electrophoresis at constant pressure, the separated protein bands were electro-transferred onto the PVDF membrane (Millipore, USA) with constant current. The membrane was then washed with PBST (0.1% Tween-20/PBS solution) three times and blocked with 5% BSA for 2 h at room temperature. Subsequently, the rabbit polyclonal anti-DOCK1 (Cat#: ab97325, Abcam, USA) and rabbit monoclonal anti-GAPDH (Cat#: EPR16891, Abcam, USA) were employed for primary antibody incubation overnight at 4 °C. HRP-conjugated Goat anti-rabbit IgG H&L (Cat#: ab181602, Abcam, USA) was used for secondary antibody incubation for 2 h at room temperature. After repeated washing with PBST, the blots were visualized using the enhanced chemiluminescence kit (Thermo Fisher Scientific, USA).

### Rac1 activation assay

This assay was performed using the Rac1 activation assay kit (Cell Biolabs, Inc., USA). Briefly, the transfected U937 and HL60 cells were collected and lysed with a prepared ice-cold lysis buffer. Then, 1 mL cell lysates were removed and incubated with 40 μL PAK PBD Agarose bead slurry at 4 °C for 1 h. After washing three times with 1 × assay buffer, the beads were resuspended in 40 µL of 2 × SDS-PAGE sample buffer and boiled for 5 min to obtain the pull-down mixture. Subsequently, the pull-down mixture was loaded into SDS-PAGE, and the protein level of Rac1 in the pull-down mixture was subjected to immunoblotting analysis.

### Statistical analysis

Three biological repeats were performed for each experiment. GraphPad Prism 8.0 (GraphPad Software, USA) was used to analyze the data and draw statistical graphs. The Student’s *t*-test and one-way ANOVA with Dunnett’s post hoc were leveraged to analyze the statistical difference between two groups and multiple groups, respectively. The Chi-squared test was employed to evaluate the correlation between the cytogenetic risk of AML patients and the expression of LINC00665 and DOCK1. The error bars in each data indicate the standard deviation (SD) of the mean. A p-value of less than 0.05 was regarded as statistically significant.

### Ethics approval and informed consent

The present study was approved by the Ethics Committee of The Fifth Affiliated Hospital of Zhengzhou University (Zhengzhou, China). The processing of clinical tissue samples is in strict compliance with the ethical standards of the Declaration of Helsinki. All patients signed written informed consent.

### Consent for publication

Consent for publication was obtained from the participants.

## Results

### LINC00665/miR-4458/DOCK1 interactome identification

LINC00665 has been reported to be a tumor promoter in various human cancers, such as lung cancer^31, 32^, gastric cancer^17, 33^, hepatocellular carcinoma^34–36^, and breast cancer^16, 37^. However, its effects on AML cells remain unexplored. Herein, we investigated the GEPIA database and found that LINC00665 was significantly upregulated in AML. This finding suggested that LINC00665 might be a tumor promoter in AML samples. In addition, we discovered a significant positive correlation between LINC00665 expression and the blast count of the AML samples collected from patients (Supplemental Fig. 1A). A correlation was also found in terms of the cytogenetic risk of AML patients (Table 3). For these reasons, LINC00665 was considered to be the key regulator in AML cells.

**Figure 1:**
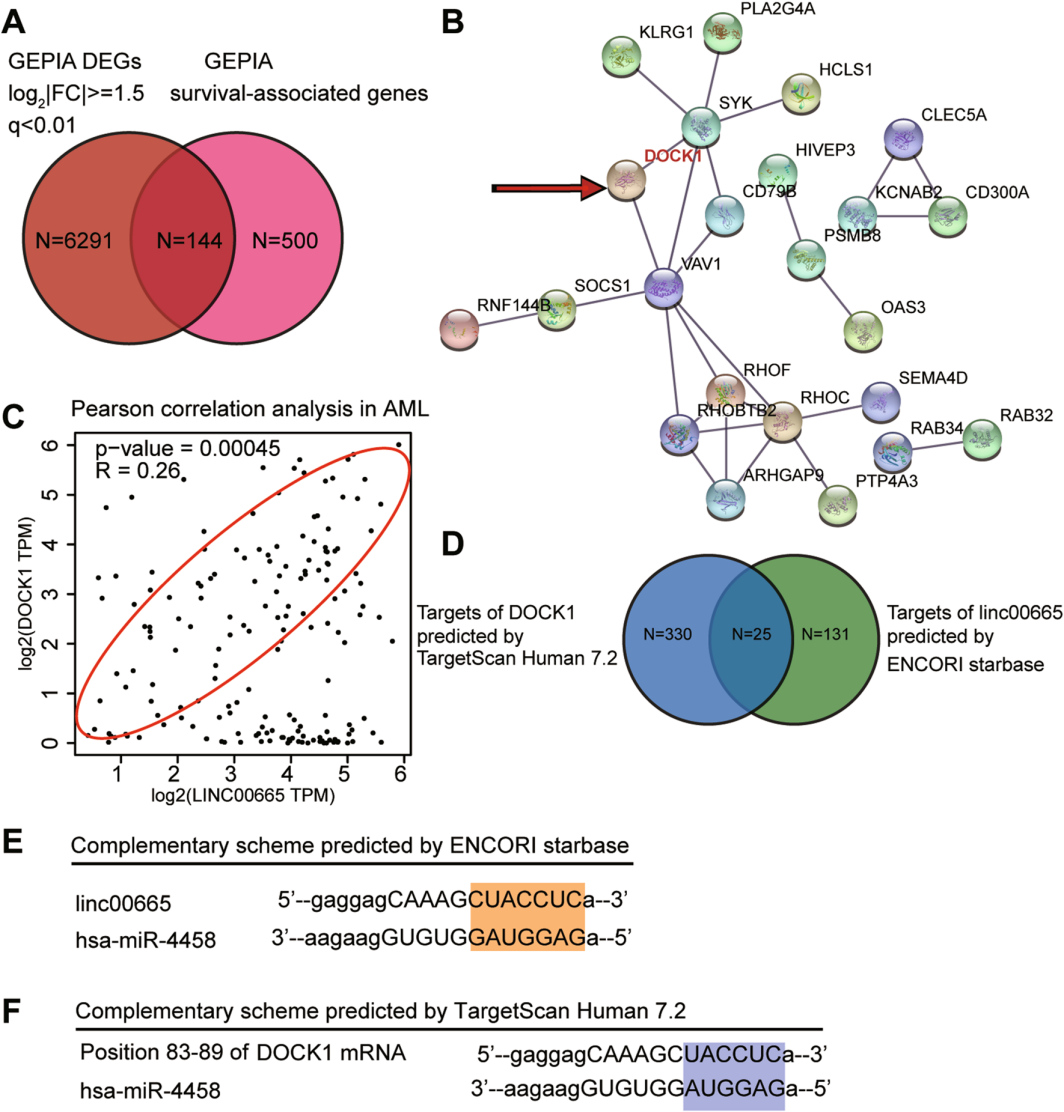
DOCK1 and miR-4458 are the key regulators in AML. (**A**) Venny 2.1.0 overlapped 144 genes from GEPIA DEGs and GEPIA survival-associated genes. (**B**) DOCK1 was identifed as the key gene by STRING analysis. (**C**) The correlation between LINC00665 and DOCK1 was analyzed using GEPIA algorithm. (**D**) 25 miRNAs bridging between LINC00665 and DOCK1 mRNA were overlapped using Venny 2.1.0. (**E**) ENCORI starBase predicted the binding site between LINC00665 and miR-4458. (**F**) TargetScan predicted the binding site between DOCK1 and miR-4458.

**Table 3:** Correlation between LINC00665 expression and cytogenetic risk of 36 cases with acute myeloid leukemia patients.

Characteristics	Total = 36	Expression of LINC00665		*P*-value^a^
		High (n = 16)	Low (n = 20)	
**Cytogenetic risk group**				0.01
Favourable	9 (25.0%)	1 (6.25%)	8 (40.0%)	
Intermediate	17 (47.2%)	7 (43.75%)	10 (50.0%)	
Unfavourable	10 (27.8%)	8 (50.0%)	2 (10.0%)	

^a^*P*‑value indicates the significance for the comparison between higher and lower LINC00665 expression (cut by the mean expression level of LINC00665) in 36 cases with acute myeloid leukemia patients.

Using the limma algorithm, 6291 DEGs (log_2_|FC|> = 1.5 and p-value < 0.01) in AML samples were obtained from the GEPIA database to identify the key gene. We also utilized GEPIA to glean 500 most significant survival-associated genes from AML tissues. By intersecting the DEGs and survival-associated genes, we found 144 genes (Fig. 1A), which were subsequently uploaded to the STRING database. DOCK1 was found to have a high confidence level (Fig. 1B). Even though DOCK1 has been reported to be a tumor promoter in a wide spectrum of human cancers, the relationship between AML and DOCK1 has not been fully explored in the literature. In addition, we identified a significant positive correlation between DOCK1 expression and the blast count of the AML samples collected from patients (Supplemental Fig. 1B). We also noticed that DOCK1 expression was positively correlated for with the cytogenetic risk of AML patients (Table 4). On top of that, the pearson correlation analysis revealed a significant relationship between LINC00665 and DOCK1 in AML tumor samples (Fig. 1C).

**Table 4:** Correlation between DOCK1 expression and cytogenetic risk of 36 cases with acute myeloid leukemia patients.

Characteristics	Total = 36	Expression of DOCK1		*P*-value^a^
		High (n = 17)	Low (n = 19)	
**Cytogenetic risk group**				0.011
Favourable	9 (25.0%)	2 (6.25%)	7 (40.0%)	
Intermediate	17 (47.2%)	7 (43.75%)	11 (50.0%)	
Unfavourable	10 (27.8%)	8 (50.0%)	1 (10.0%)	

^a^*P*‑value indicates the significance for the comparison between higher and lower DOCK1 expression (cut by the mean expression level of DOCK1) in 36 cases with acute myeloid leukemia patients.

To identify the miRNA bridging between LINC00665and DOCK1 mRNA, we utilized TargetScan Human 7.2 and the encyclopedia of RNA interactomes (ENCORI) starBase and identified 25 miRNAs (Fig. 1D). Among the top 5 ranked miRNAs, miR-4458 was screened out because it was considered to be a significant tumor suppressor in diverse cancers, excluding AML. The predicted binding relationships between LINC00665, miR-4458 and DOCK1 mRNA are illustrated in Fig. 1E,F.

### LINC00665 could directly interact with miR-4458 in AML cells

**Figure 2:**
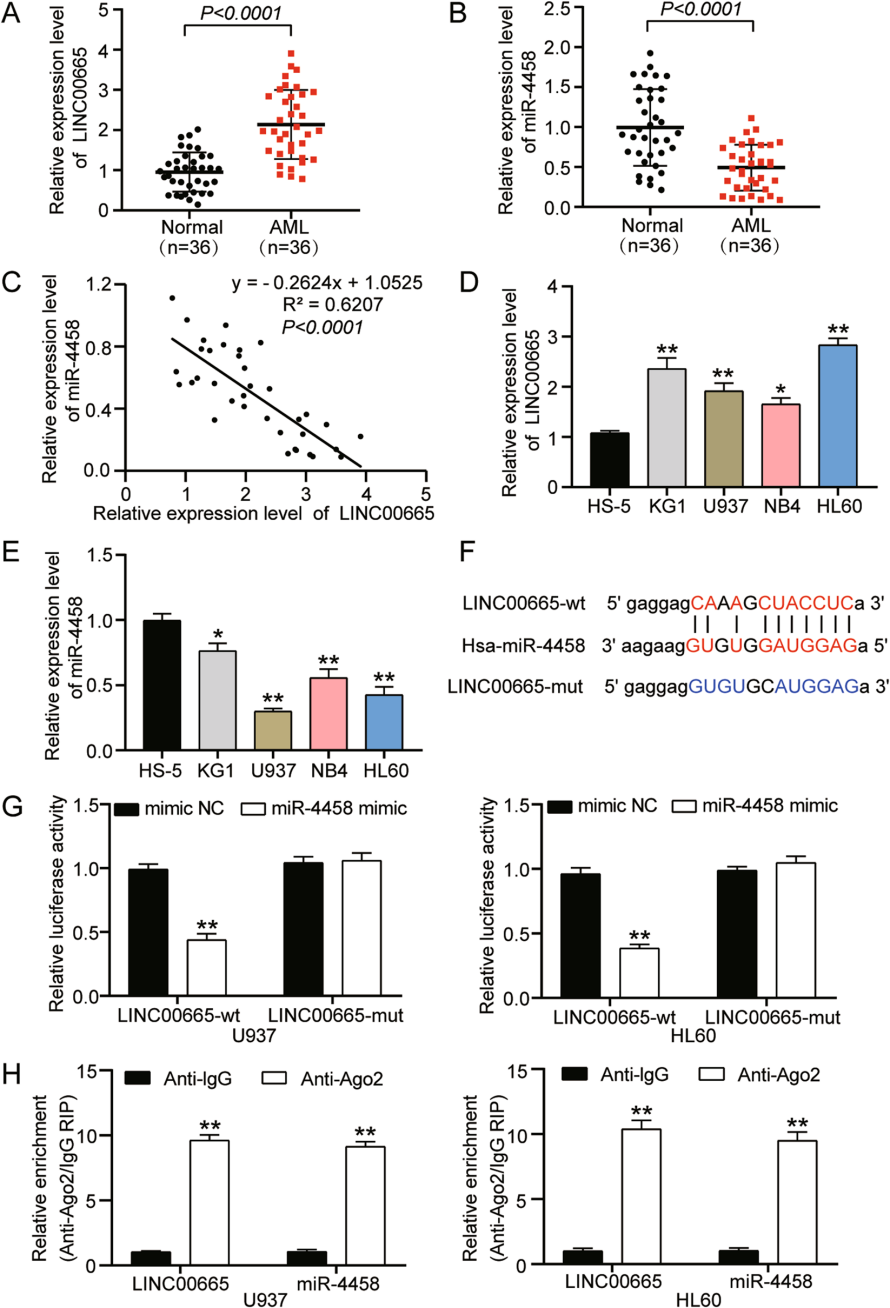
LINC00665 presents reverse expression pattern and can directly interact with miR-4458 in AML cells. (**A**) qRT-PCR analysis of relative expression of LINC00665 in AML tissues and normal bone marrow tissues. GAPDH served as reference control. (**B**) qRT-PCR analysis of relative expression miR-4458 in AML tissues and normal bone marrow tissues. U6 served as reference control. (**C**) Pearson’s correlation analysis of the correlation between the expression of LINC00665 and miR-4458 in bone marrow tissues from all donors. (**D**) qRT-PCR analysis of relative expression of LINC00665 in normal bone marrow cell line (HS-5) and AML cell lines (KG1, U937, NB4 and HL60). GAPDH served as reference control (**E**) qRT-PCR analysis of relative expression of miR-4458 in normal bone marrow cell line (HS-5) and AML cell lines (KG1, U937, NB4 and HL60). U6 served as reference control. (**F**) The miR-4458 binding site on the transcript of human LINC00665 was predicted using starBase and the mutated sequences was designed and shown. (**G**) Luciferase activity assay for U937 and HL60 cells transfected with miR-4458 mimic or negative control (mimic NC) together with luciferase reporter plasmids containing predicted (LINC-wt) or mutated (LINC-mut) miR-4458 binding sequences on LINC00665 transcripts. (**H**) RIP assay was performed to evaluate the direct interaction between LINC00665 and miR-4458. Three biological repeats were performed for each experiment, and the data were shown as mean ± SD. Statistics analysis was carried out with Student’s *t*-test or one-way ANOVA. *P < 0.05; **P < 0.001.

To determine the relationship between LINC00665 and miR-4458 in AML development, we first collected bone marrow tissues from AML patients (n = 36) and normal healthy individuals (n = 36). After that, we detected the expression level of LINC00665 and miR-4458. The qRT-PCR results displayed a twofold increase in LINC00665 and a 0.5-fold decrease in miR-4458 in the tissues of AML patients compared with that of normal individuals (Fig. 2A,B). Correlation analyses also revealed that the expression of LINC00665 was inversely correlated with miR-4458 in AML bone marrow tissues (Fig. 2C). Furthermore, the qRT-PCR analysis of AML cell lines (KG1, U937, NB4 and HL60) and normal bone marrow cell lines (HS-5) showed that LINC00665 was significantly upregulated, while miR-4458 was considerably downregulated in AML cell lines (Fig. 2D,E). These results indicated that both LINC00665 and miR-4458 might participate in AML progression.

To further investigate whether LINC00665 and miR-4458 could interact with each other, we explored their potential binding site with the ENCORI starBase algorithm and found a binding site of miR-4458 in the transcript of human LINC00665. This finding suggested that miR-4458 could interact directly with LINC00665. The predicted and mutated binding sequences are shown in Fig. 2F. We then constructed the luciferase reporter plasmids containing predicted (LINC00665-wt) and mutated (LINC00665-mut) miR-4458 binding sites on the transcript of human LINC00665. After that, we transfected the two constructs into U937 and HL60 cells, along with miR-4458 mimics or negative control, to assess the luciferase activity. The assay results showed a 0.6-fold decrease in the luciferase activity in U937 and HL60 cells co-transfected with LINC00665-wt and miR-4458 mimics compared to the LINC00665-wt construct and negative control (mimic NC). However, when the LINC00665-mut construct was transfected into U937 and HL60 cells, the luciferase activity showed no significant difference between the miR-4458 mimic group and the mimic NC group (Fig. 2G). Moreover, the RIP assay result confirmed that LINC00665 could directly bind and interact with miR-4458 in U937 and HL60 cells (Fig. 2H). Taken together, these results suggested that LINC00665 and miR-4458 could directly interact with each other in AML cells, thereby contributing to AML progression.

### LINC00665 promoted the proliferation, adhesion and migration of AML cells but restricted the apoptosis of AML cells by sponging miR-4458

**Figure 3:**
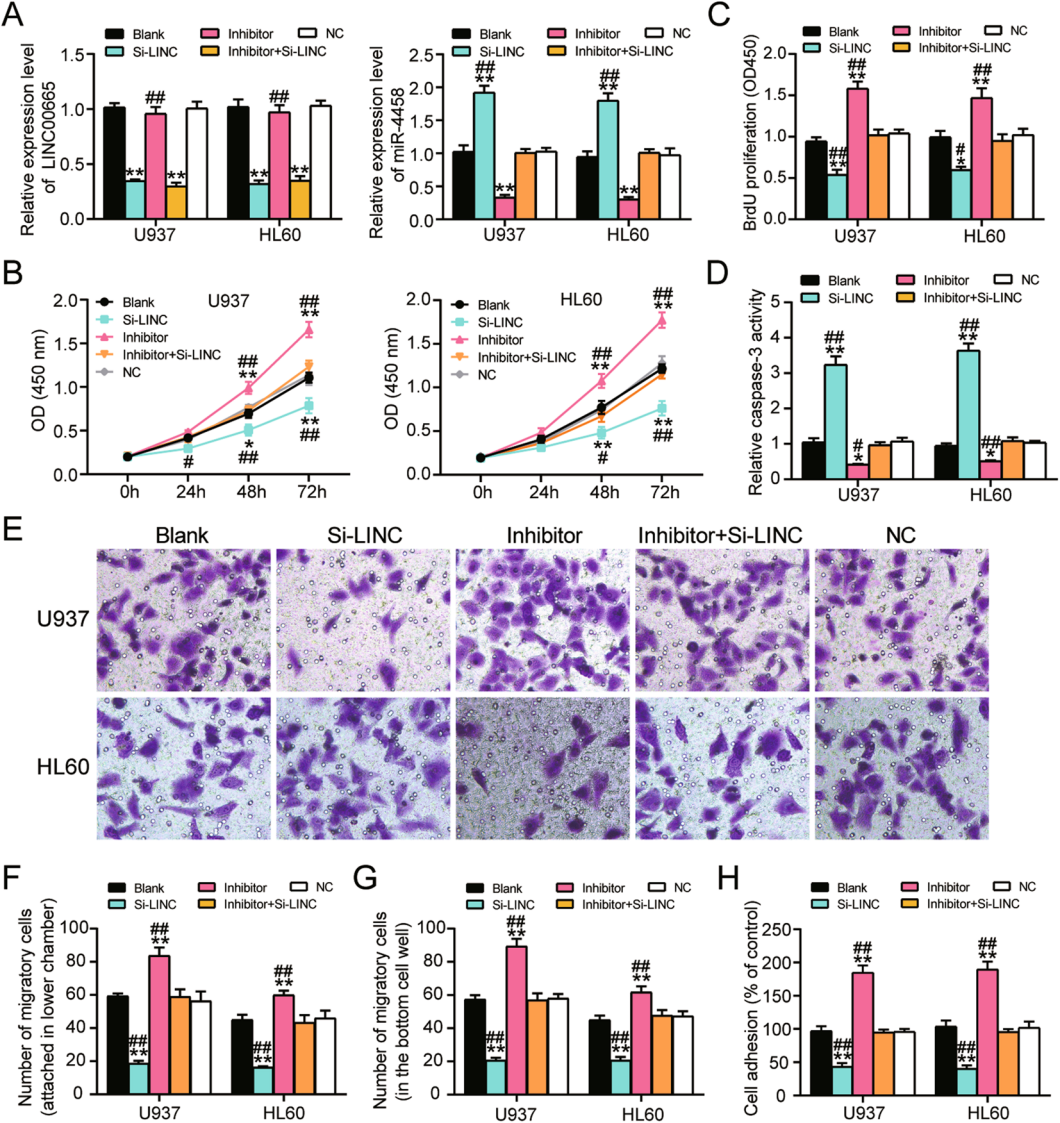
MiR-4458 promotes AML cells proliferation and adhesion and inhibit AML cells apoptosis. (**A**) qRT-PCR analysis of LINC00665 expression in U937 and HL60 cells transfected with LINC00665 siRNA (si-LINC), miR-4458 inhibitor (inhibitor), LINC00665 siRNA and miR-4458 inhibitor (inhibitor + si-LINC), negative control (NC) or cells with no treatment (Blank). (**B**) CCK-8 assay was conducted to evaluate the cell viability of U937 and HL60 cells in blank group, si-LINC group, inhibitor group, inhibitor + si-LINC group and NC group after culture for 0 h, 24 h, 48 h, 72 h. (**C**) BrdU assay was performed to determine the proliferative capacity of U937 and HL60 cells in the above groups. (**D**) Caspase-3 activity was evaluated to reflect the apoptosis of U937 and HL60 cells in the above groups. (**E**–**G**) The migration ability of U937 and HL60 cells in the above groups was evaluated by transwell assay. (**E**) Representative picture and (**F**) Statistical analysis of migratory cells attached in the bottom chamber. (**G**) Statistical analysis of migratory cells migrated into the culture medium of lower cell well. (**H**) The cell adhesion capacity of U937 and HL60 cells in the above groups was analyzed by cell adhesion assay. Three biological repeats were performed for each experiment, and the data were shown as mean ± SD. Statistics analysis was carried out with Student’s *t*-test or one-way ANOVA. *P < 0.05; **P < 0.001 versus Blank group; ^#^*P* < 0.05; ^##^*P* < 0.001 versus inhibitor + si-LINC group.

To further investigate the role of LINC00665 and miR-4458 in AML samples, we designed LINC00665 siRNA (si-LINC) and miR-4458 inhibitor (inhibitor) and transfected or co-transfected them to U937 and HL60 cells to evaluate their effect on multiple cell functions. Using qPT-PCR, we found that si-LINC led to 0.7-fold decrease in LINC00665 expression and 1.8-fold increase in miR-4458 expression compared with the negative control (NC) and untreated cells (blank). On the other hand, miR-4458 inhibitor downregulated miR-4458 expression by 0.75-fold, but it had no effect on LINC00665 expression in contrast to the NC group. After co-transfecting LINC00665 siRNA and miR-4458 inhibitor, LINC00665 expression declined in the cells, and the level of miR-4458 was comparable to that of the blank group (Fig. 3A). This finding suggested that LINC00665 siRNA and miR-4458 inhibitor could neutralize miR-4458 expression.

After CCK8 assay was used to evaluate the cell viability of U937 and HL60 cells, we noticed that the cell viability was significantly reduced in LINC00665 siRNA transfected AML cells for 48 h and 72 h. We also discovered that a high level of cell viability in the miR-4458 inhibitor after the cells were cultured for 48 h and 72 h. However, the cells co-transfected with LINC00665 siRNA and miR-4458 inhibitor indicated no significant change with the blank group (Fig. 3B). BrdU assay results also showed that the BrdU intensity was 0.5-fold lower in the si-LINC group and 1.5-fold higher in the miR-4458 inhibitor group than in the blank group even though the effect of the co-transfection was completely neutralized when co-transfecting them (Fig. 3C). This experimental outcome revealed that LINC00665 could accelerate the proliferation of AML cells by sponging miR-4458.

Furthermore, the caspase-3 activity assay findings revealed that the knockdown of LINC00665 increased the apoptosis rate of U937 and HL60 cells by threefold, while the inhibition of miR-4458 reduced the apoptosis rate of the AML cells by 0.6-fold. Besides, silencing both LINC00665 and miR-4458 could maintain the normal apoptosis rate of the two AML cells. (Fig. 3D). Although AML is a unique type of cancer that originated from hematopoietic progenitor and precursor cells, the infiltration and anchor of immature AML cells in other peripheral tissues were the most important feature of AML malignancy^2^. Thus, it is imperative to investigate the migration and adhesion abilities of AML cells^38–40^.

The transwell migration assay and adhesion assay were carried out to determine cell migration and adhesion. The results of the transwell migration assay indicated that the migratory cells attached to the lower chamber of the si-LINC group were significantly downregulated compared with the blank group, but it was approximately upregulated by fourfold in the miR-4458 inhibitor group. It was also found that the number of migratory cells attached to the lower chamber of the LINC00665 siRNA and miR-4458 inhibitor co-transfection group presented no significant difference compared with the cells in the blank group (Fig. 3E,F). The same trend was found in terms of the number of U937 and HL60 cells that migrated into the culture medium at the bottom of the cell well (Fig. 3G). The results of the cell adhesion assay in both U937 and HL60 cells were similar to those of the transwell assay, thus suggesting that LINC00665 could promote AML cell adhesion by sponging miR-4458 (Fig. 3H). Overall, these results demonstrated that LINC00665 could facilitate the proliferation, migration, and adhesion of AML cells but inhibit the apoptosis of AML cells by sponging miR-4458.

### MiR-4458 directly targeted DOCK1 and served as the mediator of LINC00665 by regulating DOCK1 in AML cells

**Figure 4:**
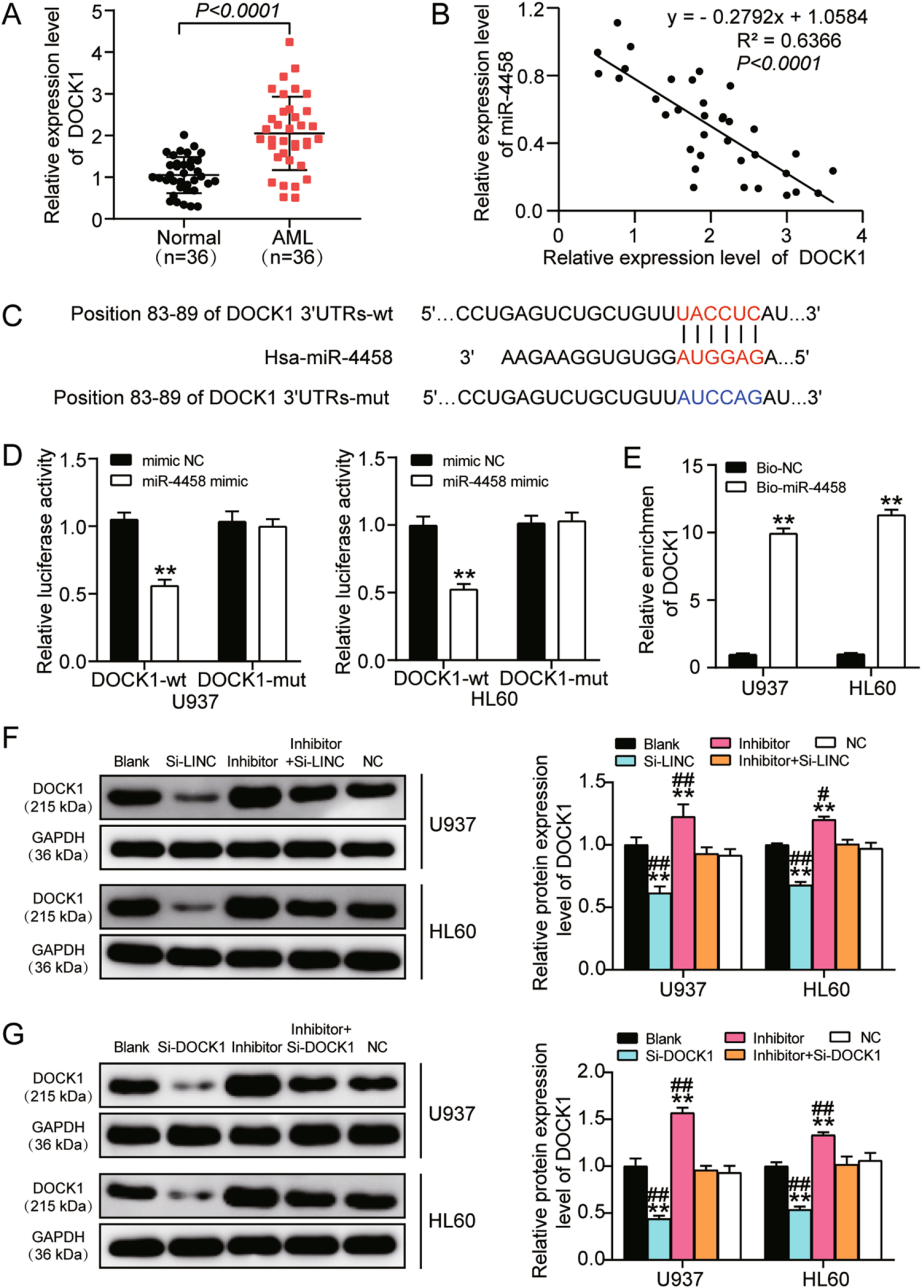
MiR-4458 directly targets DOCK1 and serves as the mediator of LINC00665 regulating DOCK1 in AML cells. (**A**) qRT-PCR analysis of relative expression of DOCK1 in AML tissues and normal bone marrow tissues. GAPDH served as reference control. (**B**) Pearson’s correlation analysis of the correlation between the expression of miR-4458 and DOCK1 in bone marrow tissues from all donors. (**C**) The miR-4458 binding site on the 3′UTR of human DOCK1 mRNA was predicted using TargetScan 7.0 and the mutated sequences was designed and shown. (**D**) Luciferase activity assay for U937 and HL60 cells transfected with miR-4458 mimic or negative control (mimic NC) together with luciferase reporter plasmids containing predicted (DOCK1-wt) or mutated (DOCK1-mut) miR-4458 binding sequences on the 3′UTR of human DOCK1 mRNA. (**E**) RNA pull-down assay was conducted to confirm the direct interaction between miR-4458 and DOCK1. (**F**) Western blot analysis of DOCK1 expression in U937 and HL60 cells transfected with LINC00665 siRNA (si-LINC), miR-4458 inhibitor (inhibitor), LINC00665 siRNA and miR-4458 inhibitor (inhibitor + si-LINC), negative control (NC) or cells with no treatment (Blank). GAPDH served as the reference control. (**G**) Western blot analysis of DOCK1 expression in U937 and HL60 cells transfected with DOCK1 siRNA (si-DOCK1), miR-4458 inhibitor (inhibitor), DOCK1 siRNA and miR-4458 inhibitor (inhibitor + si-DOCK1), negative control (NC) or cells with no treatment (Blank). GAPDH served as the reference control. Three biological repeats were performed for each experiment, and the data were shown as mean ± SD. Statistics analysis was carried out with Student’s *t*-test or one-way ANOVA. *P < 0.05; **P < 0.001 versus mimic NC group or Blank group; ^#^*P* < 0.05; ^##^*P* < 0.001 versus inhibitor + si-DOCK1 group.

To investigate the role and regulatory relationship of miR-4458 and DOCK1 in AML cells, we first measured the expression of DOCK1 in AML and normal bone marrow tissues. Our findings also revealed that DOCK1 expression was abnormally upregulated in AML tissues and that it showed a negative correlation with the expression of miR-4458 (Fig. 4A,B). After using TargetScan7.0 to predict the target gene of miR-4458, we found that DOCK1 was a potential target gene of miR-4458. To validate this predicted target relationship, we assessed the effect of luciferase reporter plasmids containing wild type (DOCK1-wt) or mutated (DOCK1-mut) miR-4458 binding sequences on DOCK1 3′-UTR. The wild type and mutated binding sequences are depicted in Fig. 4C. Subsequently, U937 and HL60 cells were transfected with the miR-4458 mimics or negative control, and the luciferase activity assay results showed that the luciferase activity decreased by 50% in DOCK1-wt and miR-4458 co-transfected AML cells. However, the DOCK1-mut and miR-4458 mimics co-transfected cells presented no significant change in the luciferase activity with DOCK1-mut and negative control co-transfected cells (Fig. 4D). Furthermore, the RNA-pull-down assay results showed that the mRNA expression level of DOCK1 in the streptavidin capture of biotinylated miR-4458 mimics (Bio-miR-4458) transfected U937 and HL60 cells was more than tenfold higher than that of the biotinylated negative control (Bio-NC) transfected cells (Fig. 4E). These data confirmed that miR-4458 could directly target DOCK1.

To assess the regulation of LINC00665 and miR-4458 to DOCK1, western blot was used to examine the protein level of DOCK1 in U937 and HL60 cells with LINC00665 silencing and/or miR-4458 inhibition. The results revealed that silencing LINC00665 resulted in a 50% decrease in DOCK1 expression and that inhibiting miR-4458 increased DOCK1 expression by 1.6-fold in AML cells. However, silencing or inhibiting both LINC00665 and miR-4458 had no effect on DOCK1 expression, meaning LINC00665 could positively regulate DOCK1 by modulating miR-4458 (Fig. 4F). Moreover, silencing DOCK1 and/or inhibiting miR-4458 in U937 and HL60 cells showed a similar effect on DOCK1 expression. This outcome further revealed the similarity between the function of the LINC00665 knockdown and the function of the DOCK1 knockdown (Fig. 4G). Taken together, these results demonstrated that LINC00665 could promote DOCK1 expression by sponging miR-4458.

### MiR-4458 hindered the proliferation, migration, and adhesion of AML cells but accelerated apoptosis of AML cells by regulating DOCK1

**Figure 5:**
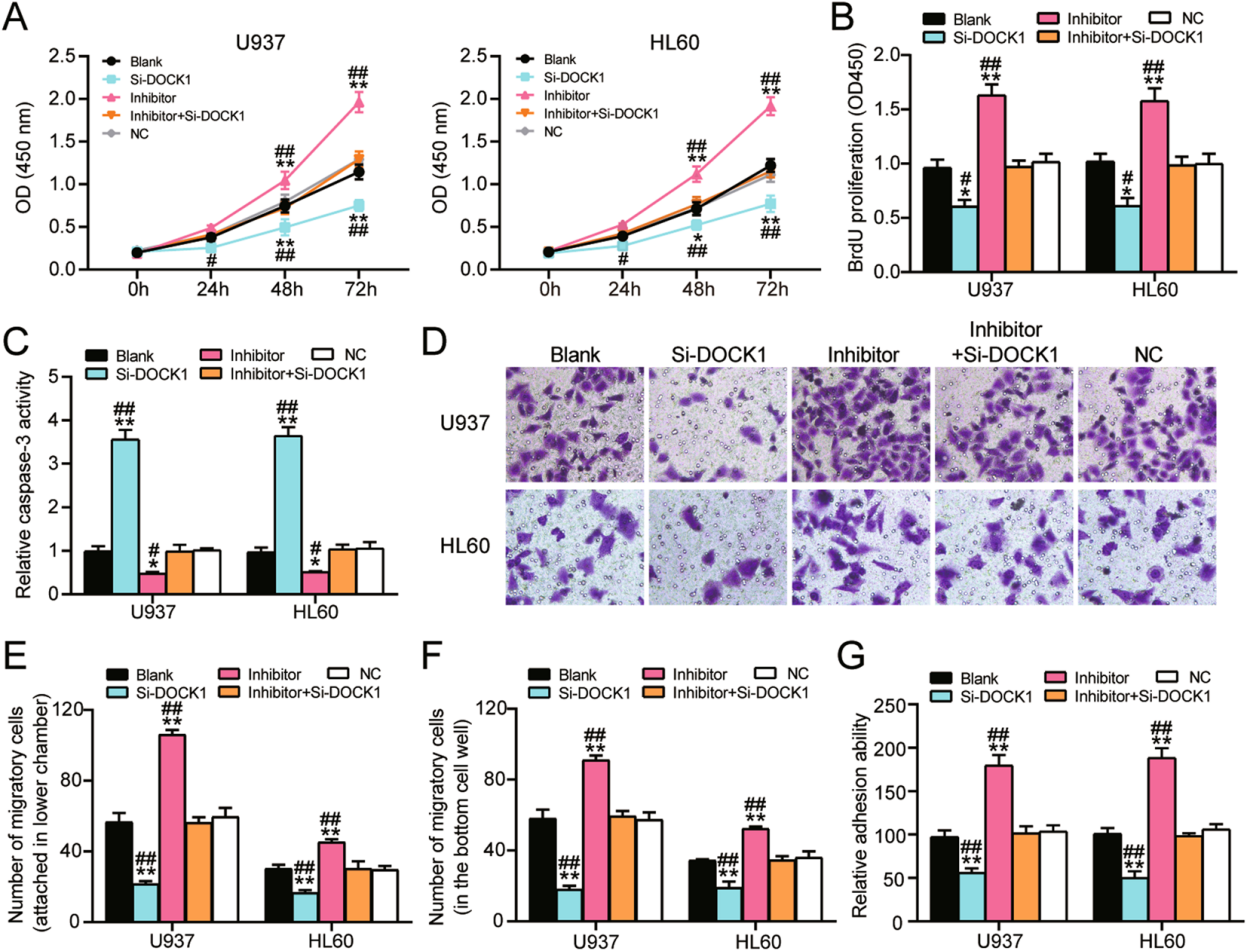
MiR-4458 exerts its accelerative effect on AML cells proliferation, migration, adhesion and restrictive effect on cell apoptosis through the regulation of DOCK1. (**A**) CCK-8 assay was conducted to evaluate the cell viability of U937 and HL60 cells transfected with DOCK1 siRNA (si-DOCK1), miR-4458 inhibitor (inhibitor), DOCK1 siRNA and miR-4458 inhibitor (inhibitor + si-DOCK1), negative control (NC) or cells with no treatment (Blank) after culture for 0 h, 24 h, 48 h, 72 h. (**B**) BrdU assay was performed to determine the proliferative capacity of U937 and HL60 cells in the above groups. (**C**) Caspase-3 activity was evaluated to reflect the apoptosis of U937 and HL60 cells in the above groups. (**D**–**F**) The migration ability of U937 and HL60 cells in the above groups was evaluated by transwell assay. (**D**) Representative picture and (**E**) Statistical analysis of migratory cells attached in the bottom chamber. (**F**) Statistical analysis of migratory cells migrated into the culture medium of lower cell well. (**G**) The cell adhesion capacity of U937 and HL60 cells in the above groups was analyzed by cell adhesion assay. Three biological repeats were performed for each experiment, and the data were shown as mean ± SD. Statistics analysis was carried out with Student’s *t*-test or one-way ANOVA. *P < 0.05; **P < 0.001 versus Blank group; ^#^*P* < 0.05; ^##^*P* < 0.001 versus inhibitor + si-DOCK1 group.

To ascertain whether miR-4458 could regulate AML cells function by targeting DOCK1, we first performed CCK assay to evaluate the viability of U937 and HL60 cells transfected with DOCK1 siRNA (si-DOCK1 group) and miR-4458 inhibitor (inhibitor group) or co-transfected with DOCK1 siRNA and miR-4458 inhibitor (inhibitor + si-DOCK group). The CCK assay results showed that the viability of U937 and HL60 cells in the si-DOCK1 group was reduced; however, this reduction was completely reversed by co-transfecting the miR-4458 inhibitor (Fig. 5A). Moreover, the BrdU assay results demonstrated that the DNA synthesis in the si-DOCK1 group declined by 40% in contrast to the blank group. It was later discovered that co-transfecting miR-4458 inhibitor and si-DOCK1 in U937 and HL60 cells showed a similar amount of DNA synthesis as the blank group. This experimental outcome suggested that miR-4458 could promote the proliferation of AML cells by influencing DOCK1 (Fig. 5B).

Furthermore, the caspase-3 activity results showed that the caspase-3 activity in DOCK1 silenced U937 and HL60 cells increased by 3.5-fold in contrast to the blank group. However, this increase was completely reversed by co-transfecting miR-4458 inhibitor (Fig. 5C). Moreover, the transwell assay indicated that the number of migratory cells attached to the lower chamber decreased significantly in the si-DOCK1 group compared with the blank group. However, this significance was not observed in the inhibitor + si-DOCK group, meaning miR-4458 inhibitor could repress the anti-migration effect of si-DOCK1 (Fig. 5D,E). The same trend was observed in the number of U937 and HL60 cells that migrated into the culture medium at the bottom of the cell well (Fig. 5F). The cell adhesion ability was finally evaluated, and results showed a reduction in the adhesion ability of DOCK1 siRNA transfected U937 and HL60 cells; however, this reduction was completely reversed by co-transfecting miR-4458 inhibitor (Fig. 5G). Overall, these results confirmed that miR-4458 could restrict the proliferation, migration, and adhesion of AML cells but that it could enhance the apoptosis of AML cells by inhibiting DOCK1.

### DOCK1 accelerated AML progression by activating Rac1

**Figure 6:**
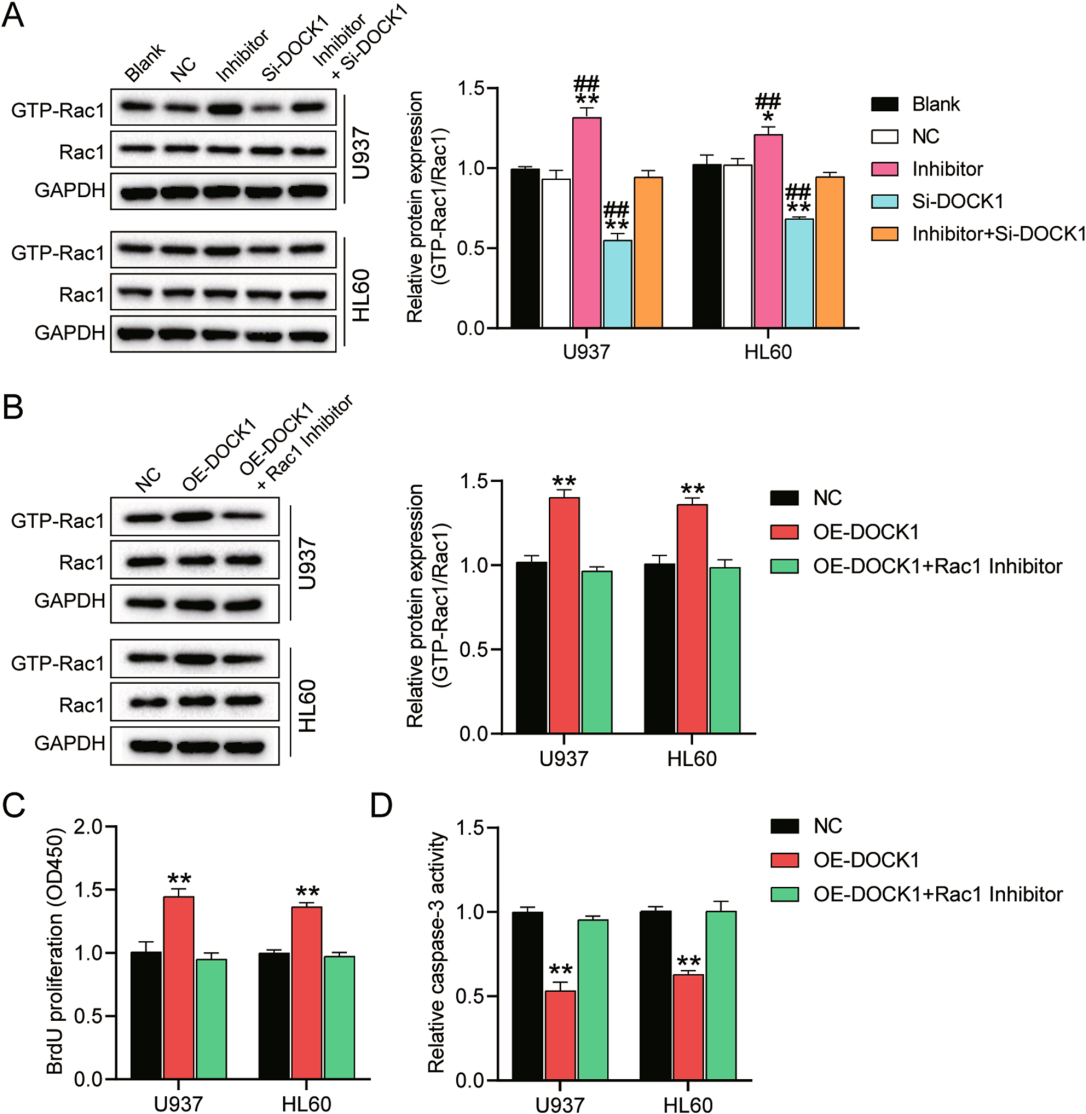
DOCK1 aggravates the AML progression through the activation of Rac1. (**A**) Western blot analysis of the relative expression of GTP-Rac1 complex in U937 and HL60 cells transfected with DOCK1 siRNA (si-DOCK1), miR-4458 inhibitor (inhibitor), DOCK1 siRNA and miR-4458 inhibitor (inhibitor + si-DOCK1), negative control (NC) or cells with no treatment (Blank). GAPDH served as the reference control. *P < 0.05; **P < 0.001 versus Blank group; ^#^*P* < 0.05; ^##^*P* < 0.001 versus inhibitor + si-DOCK1 group. (**B**) Western blot analysis of the relative expression of GTP-Rac1 complex in U937 and HL60 cells transfected with negative control (NC), DOCK1 overexpression vectors (OE-DOCK1) and/or treated with 50 μM Rac1 inhibitor. GAPDH served as the reference control. ** *P* < 0.001 versus NC group. (**C**) BrdU assay was performed to determine the proliferative capacity of U937 and HL60 cells in the above three groups. **P < 0.001 versus NC group. (**D**) Caspase-3 activity was evaluated to reflect the apoptosis of U937 and HL60 cells in the above three groups. **P < 0.001 versus NC group. Three biological repeats were performed for each experiment, and the data were shown as mean ± SD. Statistics analysis was carried out with Student’s *t*-test or one-way ANOVA.

Accumulating evidence in the literature revealed that Rac1 could function as a crucial mediator in regulating DOCK1^41, 42^. Hence, we hypothesized that Rac1 could regulate AML progression. To confirm this hypothesis, we first evaluated the abundance of the GTP-Rac1 complex, which indicated the activation of Rac1, and found that the relative abundance of the GTP-Rac1 complex increased by more than 1.2-fold in U937 and HL60 cells after AML cells were transfected with miR-4458 inhibitor. However, the abundance decreased by more than 30% when the cells were transfected with DOCK1 siRNA. More importantly, the relative abundance of the GTP-Rac1 complex was similar to that of the blank group when miR-4458 inhibitor and DOCK1 siRNA were co-transfected. This result suggested that miR-4458 could suppress the activation of Rac1 by targeting DOCK1 (Fig. 6A).

To further validate the regulation of DOCK1 by Rac1 activation, we constructed DOCK1 overexpression vectors and transfected them into U937 and HL60 cells. As shown in Fig. 6B, DOCK1 overexpression induced the upregulation (over 1.3-fold) of GTP-Rac1 complex abundance in the two AML cell lines. Nevertheless, the Rac1 inhibitor treatment effectively reversed the effect of DOCK1 overexpression on GTP-Rac1 complex abundance, meaning DOCK1 could directly promote the activation of Rac1 in AML cells (Fig. 6B). Subsequently, we investigated whether this regulatory action could affect the proliferation and apoptosis of AML cells. BrdU assay results revealed that DOCK1 overexpression enhanced DNA synthesis by 1.4-fold, but this enhancement could be completely reversed with the Rac1 inhibitor (Fig. 6C). Similarly, the caspase-3 activity results showed that the DOCK1 overexpression group decreased caspase-3 activities by 50% compared with the NC group, but this effect could be reversed with the Rac1 inhibitor (Fig. 6D). On the whole, these results demonstrated that DOCK1 could promote AML progression by activating Rac1.

## Discussion

Reports suggest that AML is the most common hematologic cancer among adults^1, 2^. Despite the technological advances in AML chemotherapy, the five-year survival rate of AML patients remains only 27%, and this unacceptable rate is mainly due to the chemoresistance and high recurrence rate of this cancer^5, 6^. Hence, it is crucial to uncover the pathogenesis of AML and explore novel strategies for AML treatment. We discovered in this research that LINC00665 and DOCK1 could promote AML development and that miR-4458 could suppress AML development. More specifically, LINC00665 could promote DOCK1 expression by targeting miR-4458 and thus contributing to AML progression.

In addition, emerging evidence has demonstrated the regulatory roles of lncRNAs in cellular processes such as cell proliferation, differentiation and apoptosis^43, 44^. These processes indicated the presence of cancers and highlighted the critical role of lncRNAs in tumorigenesis^45^. LINC00665 is a newly identified lncRNA, yet its pathophysiological role remains unknown. Although several studies indicated that LINC00665 could function as an oncogene in lung adenocarcinoma^31^, breast cancer^16^ and hepatocellular carcinoma^34^, scientists are yet to confirm its role in AML tumorigeneses. In this study, we found that LINC00665 was upregulated in AML bone marrow tissues and cell lines and found that when LINC00665 was silenced, the proliferation, migration, and adhesion abilities of AML cells were restricted, but the apoptosis ability of AML cells was enhanced. Our findings confirmed the oncogenic function of LINC00665 in AML development.

Besides, several studies have reported that lncRNAs could serve as competing endogenous RNAs (ceRNAs) that could interact with miRNAs, thereby limiting the regulatory effect on their target genes^46, 47^. Therefore, we proposed that miRNAs could be sponged by LINC00665 in AML cells. After performing bioinformatics analysis, we found that miR-4458 was an outstanding miRNA that could be regulated by LINC00665. Our starBase results further revealed the miR-4458 binding site on LINC00665 transcripts. More importantly, using luciferase reporter assay and RIP assay, we confirmed that LINC00665 could directly interact with miR-4458. While research has documented the tumor-suppressive functions of miR-4458 in multiple types of cancer^21, 23^, no studies have reported its role in the pathogenesis of AML. We herein showed the aberrant low-expression pattern of miR-4458 in AML tissues, including the promotive effects of silencing miR-4458 on AML cells’ proliferation, migration and adhesion and the inhibitory effects of silencing miR-4458 on AML cells’ apoptosis. Our experiments proved that LINC00665 exerted its tumor-aggressive role on AML cells by targeting miR-4458.

Similar to the interaction with lncRNAs, miRNAs commonly modulate their target gene expression by binding the conserved miRNAs seed sequences on the 3′-UTR of the target mRNAs, thus regulating various cellular processes. To determine the potential target gene of miR-4458, we conducted a bioinformatics analysis and found that DOCK1 was a predicted target gene of miR-4458. The results of our luciferase reporter assay and RNA pull-down assay verified this target relationship. Additionally, we demonstrated that DOCK1 was overexpressed in AML tissues, a result that was consistent with that of the previous report^29^. Moreover, we discovered that LINC00665 could regulate DOCK1 expression by sponging miR-4458 in AML cells. The subsequent functional assay results revealed that the knockdown of DOCK1 significantly reduced the proliferation, migration, adhesion and induced apoptosis of AML cells, which were regulated by miR-4458. Furthermore, we identified that DOCK1 could facilitate the proliferation of AML cells and inhibit the apoptosis of AML cells by activating Rac1. This finding was also similar to the regulatory action of DOCK1 in other types of cancer^41, 42^.

Nevertheless, this research has a few limitations. For instance, the result of this study has not been validated in vivo, and it is necessary to utilize an AML animal model to further confirm the regulatory relationship among LINC00665, miR-4458 and DOCK1. Such the animal experiment will help provide insight into the pathogenesis of AML.

## Conclusion

This research has demonstrated the role of LINC00665, miR-4458 and DOCK1 in AML progression. More specifically, our findings suggested that LINC00665 could promote the proliferation, migration, and adhesion of AML cells but inhibit the apoptosis of AML cells by sponging miR-4458 to induce DOCK1 expression, which further activated Rac1. These findings highlighted the importance of the LINC00665/miR-4458/DOCK1/Rac1 axis in the pathogenesis and treatment of AML.

## Supplementary Information

Supplementary Information

## Data Availability

The datasets used and/or analyzed during the current study are available from the corresponding author on reasonable request.

## Abbreviations

AML: Acute myeloid leukemia
lncRNAs: Long non-coding RNAs
IRF4: Interferon regulatory factor
miRNAs: MicroRNAs
DOCK1: The dedicator of cytokinesis 1
GEF: Atypical Rho guanine nucleotide exchange factors
DEGs: Differentially expressed genes
FAB: French-American-Britain
qRT-PCR: RNA extraction and Quantitative Real-time PCR
si-LINC: LINC00665 siRNA
si-DOCK1: DOCK1 siRNA
