# Supplementary material for: LINC00665 promotes the progression of acute myeloid leukemia by regulating the miR-4458/DOCK1 pathway
Source: Sci Rep. 2021 Mar 3;11:5009. doi: 10.1038/s41598-021-82834-9 (PMC7930206; doi:10.1038/s41598-021-82834-9)
Supplement: Supplementary file 1 — Supplementary Information [file 41598_2021_82834_MOESM1_ESM.docx]

Dear editor:

We submit our manuscript entitled "**LINC00665 promotes the progression of acute myeloid leukemia by regulating miR-4458/DOCK1 pathway**" for publication.

We originally declared the role of LINC00665, miR-4458 and DOCK1 in AML carcinogenesis, and elucidated that LINC00665 could promote AML cells proliferation, migration, adhesion and inhibit AML cells apoptosis by inducing the expression of DOCK1 via sponging miR-4458. These findings highlighted the importance of LINC00665/miR-4458/DOCK1 axis in AML pathogenesis and treatment.

All authors have seen the manuscript and approved to submit to your journal. This paper is new. Neither the entire paper nor any part of its content has been published or has been accepted elsewhere. It is not being submitted to any other journal.

Thank you very much for your attention and consideration.

Sincerely yours,

Xiaoyu Yang

Author list:

Xiaoyu Yang, Yan Wang, Sulei Pang, Xiaojie Li, Panpan Wang, Ruojin Ma, Yunyun Ma, Chunge Song

Supplementary Table 1. Sequences for transfection

| Name | Sequences (5’-3’) |
| --- | --- |
| miR-4458 mimic | AGAGGUAGGUGUGGAAGAA |
| mimic-NC | UUCUCCGAACGUGUCACGUUU |
| miR-4458 inhibitor | UUCUUCCACACCUACCUCU |
| inhibitor-NC | CAGUACUUUUGUGUAGUACAA |
| DOCK1 siRNA | GGACAAGCTGGACAAGGAT |
| LINC00665 siRNA | UCCUCAGUCUUGGGCUAUUTT |
| Si-NC | UUCUCCGAACGUGUCACGUTT |

Supplemental Figure 1. The expression of LINC00665 and DOCK1 has a significantly positive correlation with Blast count in 36 cases of AML patients. (A) Pearson’s correlation analysis of the relationship between LINC00665 expression and Blast count in 36 cases of AML patients. (B) Pearson’s correlation analysis of the relationship between DOCK1 expression and Blast count in 36 cases of AML patients.

DOCK1-Fig4F-HL60

DOCK1-Fig4F-U937

DOCK1-Fig4G-HL60

DOCK1-Fig4G-U937

GAPDH-Fig4F-HL60

GAPDH-Fig4F-U937

GAPDH-Fig4G-HL60

GAPDH-Fig4G-U937

GAPDH-Fig6A-HL60

GAPDH-Fig6A-U937

GAPDH-Fig6B-HL60

GAPDH-Fig6B-U937

GTP-Rac1-Fig6A-HL60

GTP-Rac1-Fig6A-U937

GTP-Rac1-Fig6B-HL60

GTP-Rac1-Fig6B-U937

Rac1-Fig6A-HL60

Rac1-Fig6A-U937

Rac1-Fig6B-HL60

Rac1-Fig6B-U937
